# The rise of artificial intelligence reading of chest X-rays for enhanced TB diagnosis and elimination

**DOI:** 10.5588/ijtld.22.0687

**Published:** 2023-05-01

**Authors:** C. Geric, Z. Z. Qin, C. M. Denkinger, S. V. Kik, B. Marais, A. Anjos, P-M. David, F. Ahmad Khan, A. Trajman

**Affiliations:** 1 Division of Experimental Medicine, Department of Medicine, McGill University, Montreal, QC, Canada; 2 Respiratory Epidemiology and Clinical Research Unit, Centre for Outcomes Research and Evaluation, Montreal, QC, Canada; 3 McGill International TB Centre, Research Institute of the McGill University Health Centre, Montreal, QC, Canada; 4 Stop TB Partnership, Geneva, Switzerland; 5 Division of Infectious Diseases and Tropical Medicine, Heidelberg University Hospital, Heidelberg, Germany; 6 German Centre for Infection Research (DZIF), partner site of Heidelberg University Hospital, Heidelberg, Germany; 7 FIND, the Global Alliance for Diagnostics, Geneva, Switzerland; 8 Sydney Medical School and Sydney Infectious Diseases Institute, The University of Sydney, Westmead, NSW, Australia; 9 Idiap Research Institute, Martigny, Switzerland; 10 Faculty of Pharmacy, Université de Montréal, Montréal, QC, Canada; 11 Departamento de Clínica Médica, Universidade Federal do Rio de Janeiro, Rio de Janeiro, RJ, Brazil

**Keywords:** computer-aided detection, chest radiology, pulmonary disease, tuberculosis, AI technology

## Abstract

We provide an overview of the latest evidence on computer-aided detection (CAD) software for automated interpretation of chest radiographs (CXRs) for TB detection. CAD is a useful tool that can assist in rapid and consistent CXR interpretation for TB. CAD can achieve high sensitivity TB detection among people seeking care with symptoms of TB and in population-based screening, has accuracy on-par with human readers. However, implementation challenges remain. Due to diagnostic heterogeneity between settings and sub-populations, users need to select threshold scores rather than use pre-specified ones, but some sites may lack the resources and data to do so. Efficient standardisation is further complicated by frequent updates and new CAD versions, which also challenges implementation and comparison. CAD has not been validated for TB diagnosis in children and its accuracy for identifying non-TB abnormalities remains to be evaluated. A number of economic and political issues also remain to be addressed through regulation for CAD to avoid furthering health inequities. Although CAD-based CXR analysis has proven remarkably accurate for TB detection in adults, the above issues need to be addressed to ensure that the technology meets the needs of high-burden settings and vulnerable sub-populations.

## Abstract (fr)

Nous fournissons un aperçu des données probantes sur les logiciels de détection assistée par ordinateur (CAD) pour l’interprétation automatisée des radiographies pulmonaires (CXR) à des fins de détection de la TB. La CAD est un outil utile qui peut contribuer à une interprétation plus rapide et cohérente des CXR dans le cadre de la TB. Elle permet de détecter la TB avec une sensibilité élevée chez les personnes consultant pour des symptômes de TB, ainsi que dans le cadre d’un dépistage en population, avec une précision comparable à celle des lecteurs humains. Cependant, des difficultés de mise en œuvre subsistent. En raison de l’hétérogénéité diagnostique entre les pays et les sous-populations, les utilisateurs doivent sélectionner des scores seuils plutôt que d’utiliser des scores préétablis mais les sites peuvent manquer des ressources et des données nécessaires pour ce faire. Une standardisation efficace est encore compliquée par les fréquentes mises à jour et les nouvelles versions de la CAD, ce qui complique également la mise en œuvre et la comparaison. La CAD n’a pas été validée pour le diagnostic de la TB chez l’enfant et sa précision pour l’identification des anomalies non tuberculeuses reste à évaluer. Un certain nombre de questions économiques et politiques doivent encore être abordées par le biais de la réglementation afin que la CAD n’aggrave pas les inégalités en matière de santé. Bien que l’analyse des CXR fondée sur la CAD se soit avérée remarquablement précise pour la détection de la TB chez l’adulte, les problèmes susmentionnés doivent être abordés afin de faciliter la mise en œuvre et de s’assurer que la technologie répond aux besoins des pays à forte prévalence de TB et des sous-populations vulnérables.

Chest radiography (CXR) is an essential tool for screening and evaluating diseases of the thorax,^1^ and for over a century it has played an important role in TB diagnosis, clinical care and follow-up. Both the availability and accuracy of CXR interpretation depend on access to skilled humans. Advances in artificial intelligence (AI) technology for radiological image analysis — also referred to as computer-aided detection (CAD) software — have led to the recent development and commercial availability of software that automate the interpretation of CXR for detecting TB, with some software also reporting on non-TB radiographic abnormalities.^2^ After undertaking a comprehensive review of AI (deep-learning) based CAD software for CXR analysis in 2021, the WHO issued a conditional recommendation that CAD solutions may be used in place of human readers for TB screening and triage in individuals aged ≥15 years.^3^ Deep artificial neural networks applied in CAD products are a ‘black-box’ algorithm with many hidden layers, which complicates assessment. Because the algorithms are optimised using a set of ‘training CXRs’, independent and peer-reviewed evaluations that apply study designs to minimise bias in estimating diagnostic accuracy of CAD products are essential.^4^ With the increased availability of CAD software for CXR analysis, a growing body of literature has shown differences in performance across products, versions and in different sub-populations. In this minireview of CAD applied to CXR, we provide an overview of evidence for TB detection and reflect on clinical, ethical and regulatory aspects related to its deployment.

## TECHNICAL CONSIDERATIONS

After analysing a CXR image, existing CAD software output a continuous ‘abnormality’ score, with higher scores indicating greater likelihood that TB is present. A threshold score must be applied to the CXR: TB is ruled out if a CXR abnormality score is below the defined threshold, whereas scores greater than the threshold indicate possible TB and usually require evaluation of a microbiological test to confirm or exclude a TB diagnosis. Although some CAD come with developer pre-specified threshold scores, CAD systems generally require users to undertake calibration studies to select the best threshold scores for their own specific setting. A generic CAD calibration protocol is available from the WHO.^5^

The main advantages and disadvantages of CAD are given in Table 1. CAD solutions can operate with portable or ultraportable X-ray machines, as well as standalone X-ray machines.^6^ Although stationary machines operated on stable electricity supply allow a high workload and deliver high image quality, portable and ultraportable systems have the advantage that they can be operated in places with intermittent power supply (ultraportables are battery-powered) and still provide good image quality with increased accessibility, albeit with a lower workload capacity.

**Table 1:** Main advantages and disadvantages of current CAD technology for CXR analysis.

Category	Advantages	Disadvantages
Technical considerations.	Can operate with both portable/ultra-portable X-ray machines and standalone X-rays	Users should calibrate CAD using local data due to known heterogeneity in the accuracy of threshold scores; only frontal images processed
Performance for TB.	Shown to perform at least on-par with radiologists	Performance can vary for different software, as well as for different versions of the same software Poorer performance has been reported for increasing age and decreasing BMI, smear-negative disease, those previously diagnosed with TB and people living with HIV; limited evidence in children Sensitivity and specificity of threshold scores vary between populations, as well as the intended application
Economic and usability aspects.	CAD has been shown to reduce costs for TB detection compared to the status quo. Improves access to TB screening Reduces turnaround time for TB diagnosis	Limited evidence; there is a need for more evidence around cost-effectiveness in different scenarios
CAD for non-TB disease.	Some CAD products have been developed for non-TB disease Performance is promising for classifying CXR as normal vs. abnormal, specifically for pneumothorax	Evidence-base for non-TB disease is limited, based on case-control studies and likely overestimating the accuracy

CAD = computer-aided detection; CXR = chest X-ray; BMI = body mass index.

## DIAGNOSTIC PERFORMANCE OF CAD FOR TB

Literature on CAD often refers to two distinct applications, or ‘use cases’ of CXR in TB detection. One is ‘triage’, where the study population consists of persons seeking care for TB symptoms. The other is ‘screening’, where the study population was not preselected by TB symptoms, but tested because they belong to a high TB prevalence (sub)population. The Supplementary Table S1 gives the main published results on CAD for TB detection. As novel CAD software and software versions are rapidly emerging, so too is evidence-base reporting on their accuracy – hence, it is important to understand potential sources of bias and challenges in applying research findings in real-world settings. Finding an optimal reference standard to assess performance has been problematic. Because human radiological interpretation is subject to inter- and intra-reader variability,^3^ studies that use human readers as the reference standard are greatly hampered by risks of bias and limited generalisability. Studies that use Xpert^®^ MTB/RIF (Cepheid, Sunnyvale, CA, USA) or culture as the reference standard are less likely to be biased. However, use of a single sputum specimen for microbiological reference testing can also result in biased estimates of CAD accuracy due to low sensitivity;^7^,^8^ this is particularly important to consider in contexts where smear-negative disease is likely to be more prevalent (screening use-case, or settings with a high prevalence of HIV co-infection). Biased estimates of CAD accuracy can also arise in studies where only participants with CXR abnormalities, whether identified using CAD or by a human reader, were selected to undergo microbiological reference standard testing.^9^ Additional gaps also exist. Most evidence relate to older adolescents and adults (individuals ≥15 years of age) who typically develop adult-type disease. Children develop a different and wider spectrum of disease, and although some commercially available CAD are marketed as being applicable for children, no performance validation in this population has been published. Another major challenge to applicability to real-world contexts is the threshold score. Selecting a threshold score is a necessary step for deploying CAD, but multi-site studies have consistently shown that the sensitivity and specificity of a given threshold score vary between populations.^10^–^12^ These differences that are partially but not fully explained by population characteristics are known to affect CAD accuracy (sex, prior TB history, age, HIV status).^10^–^12^ CAD has poorer performance with increasing age and decreasing body mass index, in smear-negative disease, in those previously diagnosed with TB and in people living with HIV.^10^,^13^–^16^ This heterogeneity in the accuracy of the threshold score means that users should calibrate CAD with local data, rather than use the reported threshold scores,^10^,^11^,^13^,^15^–^17^ and consider implementing different threshold scores for different populations, particularly for people living with HIV. This is one of the major challenges when implementing CAD: the burden on programmes to establish a threshold score with a study prior to implementation is high and prone to error, depending on how the study is done. Furthermore, thresholds vary according to the intended application of the CAD: higher sensititvity is preferred even if specificity is somehow lower when used for triage, unlike when screening is the main goal.

Large studies have evaluated commercially available deep-learning-based CAD in the triage use-case against a microbiological standard of sputum Xpert and/or culture. Three systems, qXR (Qure.ai, Mumbai, India), Lunit INSIGHT CXR (Lunit, Seoul, Korea) and CAD4TB (Delft Imaging, Delft, The Netherlands) were compared in 1,196 symptomatic participants from Nepal and Cameroon.^11^ Using Xpert as the reference standard, the products had similar performance characteristics with areas under the curve (AUC) of 0.94 (95% CI 0.92–0.97), 0.94 (95% CI 0.93–0.96), and 0.92 (95% CI 0.9–0.95) for respectively qXRv2, Lunitv4.2.7 and CAD4TBv6. When these same products were evaluated against a reference standard of two sputum cultures in 2,198 individuals from an urban tertiary hospital in Pakistan,^9^,^15^ qXRv2 (AUC 0.92, 95% CI 0.90–0.93) slightly outperformed CAD4TBv6 (AUC 0.87, 95% CI 0.85–0.88) and Lunitv3.1.0.0 (AUC 0.85, 95% CI 0.82–0.88). In comparison to the 90% sensitivity target set out by the WHO as the target product profile (TPP) for triage tests,^18^ CAD systems generally met the target compared against an Xpert reference standard, although not in all studies.^9^,^11^,^15^,^17^ The WHO recommendation was in part informed by an individual patient data-level meta-analysis pooling data from four triage studies undertaken in Pakistan, South Africa, Tanzania and Zambia, which evaluated CAD4TBv6, Lunitv3.1.0.0 and qXRv2 using 3727 CXR.^10^ Two of the studies included used at least two sputum tests as the reference, and the others used a single Xpert. Each of the three CAD products had similar AUCs with overlapping confidence intervals: CAD4TB, 0.83 (95% CI 0.82–0.84); Lunit, 0.83 (95% CI 0.79–0.86); qXR, 0.85 (95% CI 0.83–0.88), but concerns were raised about heterogeneity, in particular, the lower sensitivity for smear-negative disease and for people living with HIV. Newer commercial CAD products, such as InferRead DR Chest (Infervision, Nijmegen, The Netherlands) and JF-CXR (JF Healthcare, Eastbourne, UK), have been evaluated in a large dataset of 23,954 symptomatic individuals from Bangladesh, along with three other products.^17^ All five CAD products outperformed the local radiologists with comparable AUCs ranging from 0.85 to 0.91.

In the screening context, some studies used human CXR interpretation,^19^ or varying clinical thresholds to decide who needed to submit a sputum test.^20^,^21^ Although most studies did not assess CAD performance in true screening applications, some did. In a formal evidence review of the ‘screening use-case’ undertaken as part of the WHO guidelines process,^12^ AUCs ranged from 0.76–0.83 for CAD4TBv6, qXRv2 and Lunitv4.9.0. On comparing sensitivity to that of expert radiologists, all three CAD systems had lower specificity than the expert radiologists. Sensitivity estimates were lower when specificity was matched. In a recent evaluation of 12 CAD products for TB screening in a predominately senior population in Vietnam, seven performed on-par or better than radiologists, including Lunitv.3.1.0.0 (Lunit), CAD4TBv7 (Delft Imaging), qXRv3 (Qure.ai,), JF CXR-1v3.0 (JF Healthcare), Genkiv2 (Deeptek), ChestEyev1 (Oxpit)*,* and InferReadv1.0 (Infervision) with AUCs ranging from 0.5 to 0.82.^22^ However, this study demonstrated that some CAD products, although commercially available, performed poorly. This emphasises the need for careful scrutiny and independent performance validation of all commercial products.

Unlike traditional diagnostic tests, new CAD products frequently emerge and provide regular version updates of CAD software. The CAD products that were evaluated by the WHO, and subsequently recommended for use as an alternative to human readers,^3^ have all since been replaced by newer versions. Whether these newer versions guarantee enhanced accuracy remains to be determined. Two recent studies compared the performance of newer versions of qXR^14^ and CAD4TB^14^,^16^ with their predecessors. In both, the updated CAD versions outperformed the preceding products, demonstrating the need for continuous reassessment and objective re-validation of diagnostic accuracy and threshold calibrations. When CAD4TB versions were compared in a community-based screening programme in South Africa, the three latest versions had similar performance; however, the accuracy of triage thresholds varied between versions, further demonstrating that repeated calibration is necessary.^23^ Although regulatory agencies, such as the Food & Drug Administration, already have mechanisms to evaluate software updates, the WHO is still establishing its prequalification programme to adapt to the rapid pace of innovation in this field.

## ECONOMIC ASPECTS

For the first full economic evaluation, researchers developed a decision-analysis model to assess the cost-effectiveness of CAD as a triage tool for patients presenting with TB symptoms in a low-income, high TB incidence population in Pakistan.^24^ Compared to the status quo of smear microscopy and GeneXpert, with no prior triage, CAD as a triage tool was projected to reduce costs by respectively 19% and 37% while averting 3–4% additional disability-adjusted life-years. An active case-finding intervention using mobile CXR units equipped with CAD and Xpert testing was found to be cost-effective in Zambia, especially in populations with high HIV and TB mortality.^25^ An accurate CAD algorithm used as a triage test prior to molecular TB testing can provide 40–80% cost savings at a TB prevalence of 1–10% among the screened population.^26^ In a randomised trial among adults presenting with cough in Malawi, incorporating HIV testing in addition to CAD-based CXR analysis to triage for Xpert testing reduced time to TB treatment and untreated or undiagnosed HIV.^27^ However, no economic benefit was found.

## NON-TB DISEASE

Deep-learning algorithms analysing CXRs have also been developed for non-TB disease, including pneumonia, COVID-19 and lung cancer.^28^–^31^ There is a paucity of non-case control designed studies for evaluating the accuracy of CAD for diagnosing pneumonia, with likely overestimation of the technology’s accuracy.^29^ Of the certified commercial products available for TB detection using CXR, 8 out of 9 also report on non-TB findings. Three have published results where computed tomography (CT) scans were used to define the reference standard.^2^ Again, the current evidence-base on case-control studies probably overestimate CAD accuracy. To note, it has been reported that CAD CXR reading correlate better with human radiologist reading of the CXR than when CT-based CAD is used as the reference standard.^32^ Although evaluations for non-TB findings remain limited, certain software do show promise for differentiating between normal and abnormal CXR, particular for pneumothorax.^33^–^35^

## ETHICAL AND REGULATORY ASPECTS

The ethical and regulatory aspects, including the current economic and political barriers to greater implementation of CAD are outlined in Table 2. WHO approval of CAD for TB screening paved the way for the regulation of a rapidly developing global market. There are now 17 CAD competitors on the market,^2^ but only two are listed in the Stop TB Partnership’s Global Drug Facility catalogue,^36^ the leading global procurement and supply mechanism for TB devices. However, efforts are underway to update and expand the catalogue. The WHO prequalification process for these technologies is still under discussion and the regulatory needs are significant, particularly in relation to the technical, economic and political issues.^37^ Indeed, many developers emphasise their compliance with certification, but this does do not completely guarantee the quality of the product. Furthermore, given the rapid pace of software development, regulation is especially important for CAD. As more accurate versions are released, health equity could be compromised if these are not made available to all users. Regulations should also be enacted to address important technical aspects such as calibration. And there is a need to define acceptable business models for companies listed on financial markets in wealthy countries for a technology mainly targeting resource-limited countries with high TB incidence rates. Another important issue is the sovereignty of states and patients over their data when the data economy is increasingly a central part of the valuation and legitimacy of global health programmes. Additional concern may be the security of potentially sensitive health data stored in the cloud, a feature of most CADs.

**Table 2:** Economic and political barriers to greater adoption of CAD.

Issue	Barriers
There is a need for agreement on CAD specifications and regulation.	There are only two CAD products listed in the Stop TB Partnership’s Global Drug Facility catalogue, although there are currently 17 CAD competitors on the market The WHO prequalification process for CAD is still under debate Certifications that most CAD developers comply with do not guarantee the quality of the products New CAD software and new versions of current software are rapidly developing, leading to concerns around health equity if more accurate versions are not made available to all users CAD results are often based on the selection of a threshold score, which can be difficult as it should be calibrated using local data
Acceptable business models should be defined.	CAD is primarily deployed in resource-limited and high TB burden countries but developed by companies listed on financial markets in wealthy countries There is limited evidence on cost-effectiveness of CAD
Preventing misuse of data.	Authority of states and patients over their own data is of concern, as the data economy is becoming more important in the evaluation and legitimacy of global health programmes Many CAD software offer analysis via data stored in clouds, raising concerns around the security of potentially sensitive health information

CAD = computer-aided detection.

## CONCLUSION

CAD is a powerful tool with the potential to improve access to TB screening and reduce the turnaround time for TB diagnosis, but further refinement and better standardisation is needed. First, it would be ideal if CADs were able to detect non-TB lung diseases as well, since most patients seeking care with respiratory symptoms may not have TB. Second, better accuracy for TB screening in sub-populations is needed. CAD systems can tailor their performance by using local data through a continuous learning process, but this needs to be validated and at present most CAD products do not attain WHO’s TPP for triage. Moreover, CAD products have not been validated for TB screening or triage in children. Paediatric studies are hampered by the lack of a reliable microbiological and composite reference standard for children with paucibacillary disease and a wide disease spectrum. The addition of a lateral CXR to assess hilar adenopathy (currently CADs read only anteroposterior CXR) and the use of relevant clinical information may further improve accuracy.^38^ Additional issues identified from an assessment of users’ perspectives in 19 countries include the need for clearer thresholds for decision-making and for connectivity and integration with local health information systems.^39^ More evidence on cost-effectiveness and best implementation strategies, as well as the population-level impact of implementing CAD screening, is also needed. Given the variety of commercial products available and entering the market, together with regular updates of these products, careful review and standardised assessment are needed before making long-term procurement decisions.
